# Sex-Specific Exposure–Effect Relationship Between Physical Activity and Incident Atrial Fibrillation in the General Population: A Dose–Response Meta-Analysis of 16 Prospective Studies

**DOI:** 10.3389/fcvm.2021.710071

**Published:** 2021-09-22

**Authors:** Qin Wan, Yue Zhou, Wengen Zhu, Xiao Liu

**Affiliations:** ^1^Department of Geriatrics, Jiangxi Provincial People's Hospital Affiliated to Nanchang University, Nanchang, China; ^2^State Key Laboratory of Ophthalmology, Zhongshan Ophthalmic Center, Sun Yat-sen University, Guangzhou, China; ^3^Department of Cardiology, The First Affiliated Hospital of Sun Yat-sen University, Guangzhou, China; ^4^Department of Cardiology, Sun Yat-sen Memorial Hospital of Sun Yat-sen University, Guangzhou, China; ^5^Guangdong Province Key Laboratory of Arrhythmia and Electrophysiology, Guangzhou, China

**Keywords:** atrial fibrillation, physical activity, risk factor, dose-response, meta-analysis

## Abstract

**Background:** Since evidence regarding the relationship between physical activity (PA) and atrial fibrillation (AF) incidence is inconsistent among studies, we performed a dose–response meta-analysis to comprehensively evaluate the exposure–effect association between PA and incident AF and the potential sex difference in the general population.

**Methods:** The PubMed and Embase databases were searched for eligible studies published up to July 2020 (PROSPERO: CRD42018091692). The non-linear or linear exposure–effect relationship between PA and AF was examined using the robust error meta-regression method.

**Results:** A total of 16 prospective studies involving 1,449,017 individuals and 39,884 AF cases were included. We observed an inverse non-linear association between PA level and incident AF (*I*^2^ = 0%, *p*_non−linearity_ < 0.001). In the linear model, a 5 metabolic equivalent of task (MET)-h/week increase in PA was associated with a decreased risk of AF [risk ratio (RR) = 0.992, 95% confidence interval (CI): 0.988–0.996, *I*^2^ = 0%]. In the sex-stratified analysis, we observed an inverse non-linear relationship between PA level and AF risk in females (*I*^2^ = 90%, *p*_non−linearity_ < 0.0001) but not in males (*I*^2^ = 0%, *p*_non−linearity_ = 0.40). In the linear model, a 5 MET-h/week increase in PA was associated with a reduced risk of AF in females (RR = 0.982, 95% CI: 0.975–0.989, *I*^2^ = 71%) but not in males (RR = 0.998, 95% CI: 0.994–1.002, *I*^2^ = 0%), with a significant interaction observed between the two groups (*p*_interaction_ < 0.0001).

**Conclusion:** There was an inverse non-linear relationship between PA level and incident AF in the general population. The beneficial effect of PA in reducing AF risk might be predominantly observed in females.

## Introduction

Atrial fibrillation (AF) is the most common cardiac arrhythmia encountered in clinical practice, and it is associated with increased risks of stroke or systemic thromboembolism, mortality, and disability. Although the etiology of AF is still not fully understood, several modifiable risk factors, such as obesity, smoking, hypertension, alcohol abuse, and excessive exercise, have been identified (1, 2). Physical activity (PA) is defined as any movement that is produced by skeletal muscular action and results in energy consumption. Whether interventions targeting volumes of PA could be an effective strategy in the management of AF needs further investigation.

Previously, a series of studies examined the association of PA with AF risk in the general population but yielded conflicting results. Valenzuela et al. concluded that long-term endurance exercise was associated with an increased risk of AF among competitive athletes, whereas in non-athletes, either total PA or intense PA did not influence AF incidence (3). The neutral results observed in non-athletes could be partly explained by the sex difference: increased PA is associated with a decreased risk of AF in females but not in males (4). In addition, the association between PA and AF risk should be interpreted with caution because of the methodological limitations of existing studies (3). Moreover, a recent study by Elliott et al. examined a large prospective cohort of 402,406 adults and found an inverse association between total PA volume and AF risk (5), inconsistent with previous reports of a U-shaped or J-shaped association of PA with AF risk. Considering the emerging evidence and controversial findings (5–8), the association of PA level with AF risk warrants further evaluation. Furthermore, given our previous findings regarding discrepant effects of physical activity among males and females (4). We performed a dose–response meta-analysis to comprehensively investigate the exposure–effect association of PA levels with AF incidence in the general population, as well as potential sex-dependent effects.

## Methods

This meta-analysis was performed in accordance with the guidance from the Cochrane Handbook for Systematic Reviews. The results are presented in accordance with the Preferred Reporting Items for Systematic Reviews and Meta-analyses (PRISMA) guidelines (Supplementary Table 1). We did not obtain ethical approval because only previously published studies were included in this analysis. The data that support the findings of our study are available from the corresponding author upon reasonable request. The protocol of this meta-analysis has been registered with the PROSPERO International Prospective Register of Systematic Reviews (CRD42018091692).

### Literature Search

The PubMed and Embase electronic databases were systematically searched for eligible studies that reported the relationship between PA and incident AF in the general population until July 2020. Two kinds of keywords and their similar terms were applied in the search: (1) “*physical activity*” OR “*exercise*,” AND (2) “*atrial fibrillation*” OR “*atrial flutter*.” To reduce the possibility of missing retrievals, we checked the reference lists of our included studies as well as that of a newly published umbrella review (3). We applied no language restrictions in the search.

### Inclusion and Exclusion Criteria

Studies were considered eligible if they (1) performed a *post-hoc* analysis of randomized clinical trials (RCTs) or observational prospective cohort studies; (2) reported the relationship between leisure-time PA or total PA and incident AF in the general population; (3) provided adjusted risk estimates [risk ratios (RRs) and corresponding 95% confidence intervals (CIs)]; and (4) reported the PA dose in units of metabolic equivalent of task (MET)-h/week or other measures that could be used to calculate the values for analysis.

We excluded studies that assessed the effect of PA on AF risk in competitive athletes (long-term endurance exercise) who compete at different levels. We also excluded studies that focused on work-related PA. Since PA and physical fitness have different definitions and prognostic effects, studies that determined the association between physical fitness and AF were excluded. Abstracts, reviews, editorials, letters, animal studies, and studies with insufficient data were excluded. In cases of multiple reports based on the same data source, we used the risk estimates from the study with the longest follow-up or the largest sample size.

### Data Extraction

Two investigators independently reviewed the titles and abstracts of the search records to identify potential studies according to the predefined inclusion criteria. We obtained more detail by screening the full texts of the retrieved studies. Disagreements were resolved by consensus or discussion with a third investigator. Relevant data were collected, including baseline characteristics of the population (e.g., study design, data source, participant age, participant sex, and sample size), diagnosis of AF, AF cases, follow-up duration, measurement of PA, unit of PA assessment (e.g., kilocalories per week, MET-hours per week, minutes per week, and kilometers per hour), covariates in the multivariable models, and adjusted RRs and 95% CIs in each category of PA. If two or more adjusted RRs were reported in a study, the most complete RR was collected.

### Quality Assessment

The Newcastle-Ottawa Scale (NOS) (9) was used to evaluate the quality of the observational cohort studies. In *post-hoc* analyses, RCTs can be treated as cohorts when assessing study quality (10). This scale has three domains with a maximum possible score of 9 points: the selection of cohorts (0–4 points), the comparability of cohorts (0 to 2 points), and the assessment of outcome (0–3 points). An NOS score of ≥6 was considered to indicate moderate to high quality; otherwise, it was considered to indicate low quality (11, 12).

### Exposure Quantification

Considering that the PA units varied across the included studies, we quantified the group-level exposure estimates using the common unit of METs (h/week), allowing for the integration of different intensities or durations of PA exposure. To assign specific intensity to PA exposure, we regarded 3, 4, 4.5, and 8 METs as the mean intensity for light PA, moderate PA, moderate to vigorous PA, and vigorous PA, respectively (13) (https://www.who.int/dietphysicalactivity/physical_activity_intensity/en/). The intensity of the marginalized PA dose (MET-h/week) was considered in the sensitivity analysis by subtracting the resting metabolic rate of 1 MET from the raw mean PA intensity (2, 3, 3.5, and 7 METs for light PA, moderate PA, moderate to vigorous PA, and vigorous PA, respectively) (14, 15). In addition, we conducted transformations between kcal/week (Y) and MET-h/week (X) using the following formula (16):


$$\begin{array}{l} \frac{X\;\left[Met*h\right]}{Y\;\left[kcal\right]}=\frac{4.5\;\left[Met\right]*2.5\;\left[h\right]}{550\;\left[kcal\right]} \end{array}$$

When the PA dose (MET-h/week) was not directly reported in some included studies, we multiplied the median or midpoint duration of the reported category by its assigned MET value. If PA was reported only as a frequency (sessions/week), a single session was assumed to be the estimated mean duration (45 min) in the primary analysis (14, 15). An assumed duration of 30 min per session was used in the sensitivity analysis (14, 15). An overview of the MET-h/week dose-assignment calculations (both in the primary analysis and sensitivity analysis) is presented in Supplementary Tables 2, 3. For studies that reported PA levels at different ages, we only used PA exposure at the latest time.

### Statistical Analyses

The heterogeneity across the included studies was assessed using *I*^2^ statistics, where 25–50%, 50–75%, and >75% indicated low, moderate, and high heterogeneity, respectively (17). A random effects model was used to pool the risk estimates. Robust error meta-regression methods were used to fit the possible non-linear or linear relationships (18). The methods required at least two levels of PA and the corresponding RRs with variance estimates. If a study did not use the lowest PA group as the reference, the data were transformed using the methods of Hamling et al. (19). In this situation, the number of AF cases and participants in each category of PA were required. If a study did not provide the median or mean PA in each category of PA, we estimated the midpoint of each category by averaging the lower and upper boundaries of that category (12). If the highest or lowest category was open-ended, we assumed that the open-ended interval length was the same as that of the adjacent interval (12). We used 5 MET-h/week in our linear analysis, defined according to the baseline dose PA levels recommended by WHO (150–300 min of moderate-intensity PA, range ≈5–111.5 MET-h/week). Subgroup analyses were performed based on age, measurement of PA, region, follow-up duration, sample size, and AF diagnosis. Publication bias was investigated by funnel plots for a visual inspection of asymmetry and statistically assessed by Egger's and Begg's tests.

Statistical analyses were performed using Review Manager software (version 5.3, the Cochrane Collaboration 2014; Nordic Cochrane Center Copenhagen, Denmark) and Stata software (Version 14.0, Stata Corp. LP, College Station, Texas, USA).

## Results

### Study Selection

**Figure 1:**
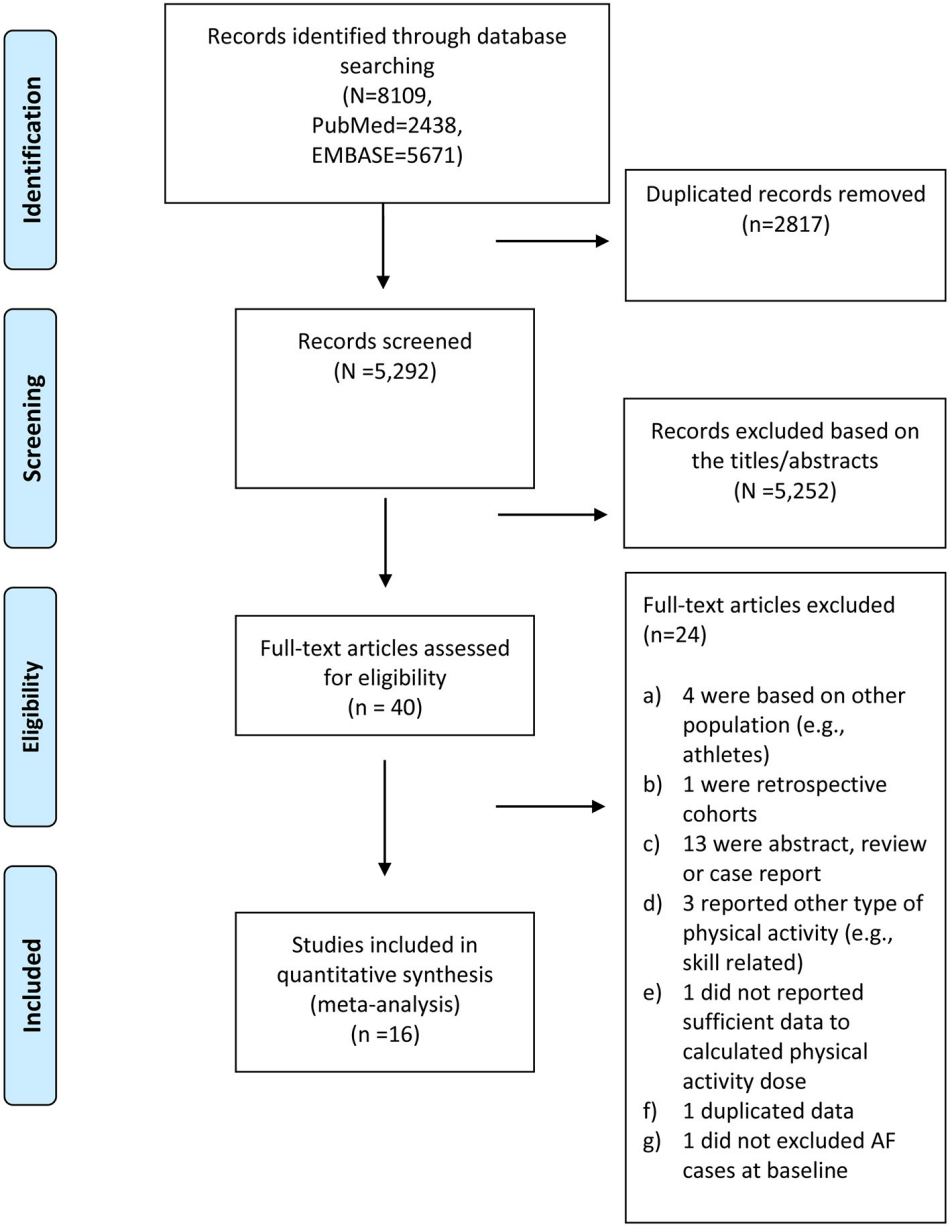
Flow chart of study selection for our dose–response analysis.

The process of study selection is shown in Figure 1. We initially identified 8,109 records in the electronic PubMed and Embase databases. After reviewing the titles and abstracts of the search records, the full texts of 40 retrieved studies were reviewed for detailed evaluation. We excluded 24 studies because they (1) assessed the association of PA with AF in competitive athletes (long-term endurance exercise); (2) were designed as retrospective cohorts or case–control studies; (3) were conference abstracts, reviews, or case reports; or (4) focused on duplicate populations. Finally, we included a total of 16 prospective studies [1 *post-hoc* analysis of RCTs (20) and 15 prospective cohorts (5–8, 21–31)] representing a total sample size of 1,449,017 individuals and 39,884 AF cases.

### Study Characteristics

A summary of the study characteristics is provided in Table 1 and Supplementary Table 4. Among the included studies, the sample size ranged from 5,446 to 501,690, and the cumulative AF incidence ranged from 0.14 to 19.48%. The mean age of the study population ranged from 38.0 to 72.8 years. The duration of follow-up ranged from 3.7 to 22 years. Nine studies (5, 7, 8, 22, 25, 26, 29–31) and 11 studies (5, 7, 8, 20, 21, 23, 25, 26, 29–31) reported the association between PA and AF in males and females, respectively. Apart from the study by O'Neal et al. (8), the studies relied on self-reported PA collected by questionnaire or interview. Regarding AF measures, those of 13 studies were based on electrocardiogram or codes of the International Classification of Diseases; 2 studies (8, 31) used electrocardiogram and/or self-reported medical history, and 1 study (29) applied the prescription of flecainide or sotalol. Each of the included studies was moderate-to-high quality, as indicated by their NOS quality scores of >7 (Supplementary Table 5).

**Table 1:** A summary of basic characteristics of the included studies.

**References, country**	**Source of individuals**	**Follow-up (years)**	**Sex**	**Mean age (years)**	**AF diagnosis**	**Case/sizeAF prevalence (%)**	**Measurement of PA**	**Adjusted covariates**
Lee et al. (6), Korea	Kangbuk Samsung Health Study	5.6	M/W	44.7	ECG	304/211,992(0.14%)	Questionnaire	Age, sex, center, year of screening examination, smoking status, alcohol intake, and education level. BMI, DM, hypertension, cardiovascular disease, and hs-CRP
Choi et al. (31), Korea	Korean Genome and Epidemiology Study	12.0	M/W	50.0	ECG or self-reported history of physician-determined diagnosis	167/8,811(1.89%)	Questionnaire	Age, sex, residence, education, BMI, comorbidity, alcohol, and smoking, and RHR
Jin et al. (7), Korea	NHIS cohort of Korean	4.0	M/W	47.6	ICD	3,443 /501,690(0.68%)	Questionnaire	Age, sex, BMI, HF, hypertension, DM, previous MI, prior stroke or transient ischemic attack, chronic kidney disease, smoking, and alcohol drinking
Elliott et al. (5), UK	UK Biobank cohort	7.0	M/W	56.0	ICD	8,640/402,406(2.14%)	Questionnaire	Age, BMI, smoking, alcohol intake, hypertension, Type 2 DM, sleep apnea, HF, valvar disease, and coronary artery disease
Albrecht et al. (30), USA	Rotterdam Study	16.9	M/W	69.4	ECG	800/7,018(11.39%)	Questionnaire	Age, sex, all other PA types, smoking, previous CVDs, alcohol consumption, diet, education, BMI, total and HDL, DM, lipid reducing agents, SBP, DBP, anti-thrombotic agents, and ACE-inhibitor use.
O'Neal et al. (8), USA	REGARDS Study	3.5	M/W	63.0	ECG and self-reported medical history	439/5,147(8.52%)	Objectively Measured	Age, gender, race, education, income, and geographic region, with the addition of SBP, HDL, total cholesterol, BMI, smoking, DM, antihypertensive medications, LVH, and previous cardiovascular disease
Mokhayeri et al. (25), USA	MESA Study	11.0	M/W	62.3	ICD	242/6,487(3.73%)	Questionnaire	Race, income, pack-years of smoking, BMI, alcohol, HP, lipids (total cholesterol, HDL), and diabetes
Morseth et al. (26), Norway	Tromsø Study survey	20.0	W/M	38.0	ECG	750/20,484(3.66%)	Questionnaire	Age, sex, BMI, height, daily smoking, CVD, SBP, DBP, DM, hypertension treatment, RHR, and use of heart medication
Skielboe et al. (28), Denmark	Copenhagen City Heart Study	20.3	M/W	48.0	ECG or ICD	1,192/17,196(6.93%)	Questionnaire	Age, height, BMI, sex, smoking, drinking habits, school education, BP, RHR, spirometry, cardiac medication, DM, ischemic heart disease, and enrolment number
Drca et al. (23), Sweden	Swedish Mammography Cohort	12.0	W	60.0	ICD	2,915/36,513(7.98%)	Questionnaire	Age, education, smoking status, pack years of smoking, BMI, DM, history of hypertension, history of CHD or HF, family history of MI, aspirin use, and alcohol consumption
Azarbal et al. (21), USA	WHI Observational Study	11.5	W	64.5	ICD	9,792/81,317(12.04%)	Questionnaire	Age, race, education, BMI, hypertension, DM, hyperlipidemia, CHD, HF, PAD, and smoking
Drca et al. (22), Sweden	People in Västmanland and Örebro	12.0	M	60.0	ICD	4,568/44,410(10.28%)	Questionnaire	Age, education, smoking status, pack years of smoking, BMI, diabetes, history of hypertension, history of CHD or HF, family history of MI, aspirin use, and alcohol consumption
Huxley et al. (24), USA	ARIC Study	22.0	M/W	54.2	ECG	1,775/14,219(12.48%)	Questionnaire	Age, race, study site, education, income, prior CVDs, cigarette smoking, height, and alcohol consumption
Thelle et al. (29), Norway	People in Norwegian	5.0	M/W	42.5	Prescription of flecainide or sotalol	863/309,540(0.27%)	Questionnaire	Age, year of screening, education, BMI, height, daily smoking, self-reported CVDs at screening, and dispensed CVDs drug
Everett et al. (20), USA	Women's Health Study	14.4	W	54.6	ECG	968/34,759(2.78%)	Questionnaire	Age, randomized treatment, cholesterol, current smoking, past smoking, alcohol, DM, race, hypertension, and BMI
Mozaffarianet et al. (27), USA	CHS Study	12.0	M/W	72.8	ECG	1,061/5,446(19.48%)	Questionnaire	Age, sex, race, enrollment site, education, smoking status, pack-year of smoking, CHD, chronic pulmonary disease, DM, alcohol use, and β-blocker use

*PA, physical activity; AF, atrial fibrillation; CI, confidence interval; MET-h/wk, the metabolic equivalent of task-h/week; M, men; W, women; ECG, electrocardiogram: relativ;e risk; ICD, International Classification of Diseases; BMI, body mass index; CVD, cardiovascular disease; CHD, Coronary heart diseases; RHR, resting heart rate; DM, diabetes mellitus; CHD, coronary heart disease; HF, heart failure; MI, myocardial infarction; PAD, peripheral artery disease; BP, blood pressure; SBP, systolic blood pressure; DBP, diastolic blood pressure; HDL, high-density lipoprotein cholesterol; LVH, left ventricular hypertrophy; hs-CRP, high sensitivity C-reactive protein; MESA, Multi-Ethnic Study of Atherosclerosis; CHS, Cardiovascular health study; ARIC, Atherosclerosis Risk In Communities; WHI, Women's Health Initiative; SCHPPM, Seirei Center for Health Promotion and Preventive Medicine; Korean National Health Insurance Service (NHIS); REGARDS, Reasons for Geographic and Racial Differences in Stroke*.

### Dose–Response Association Between PA and AF

**Figure 2:**
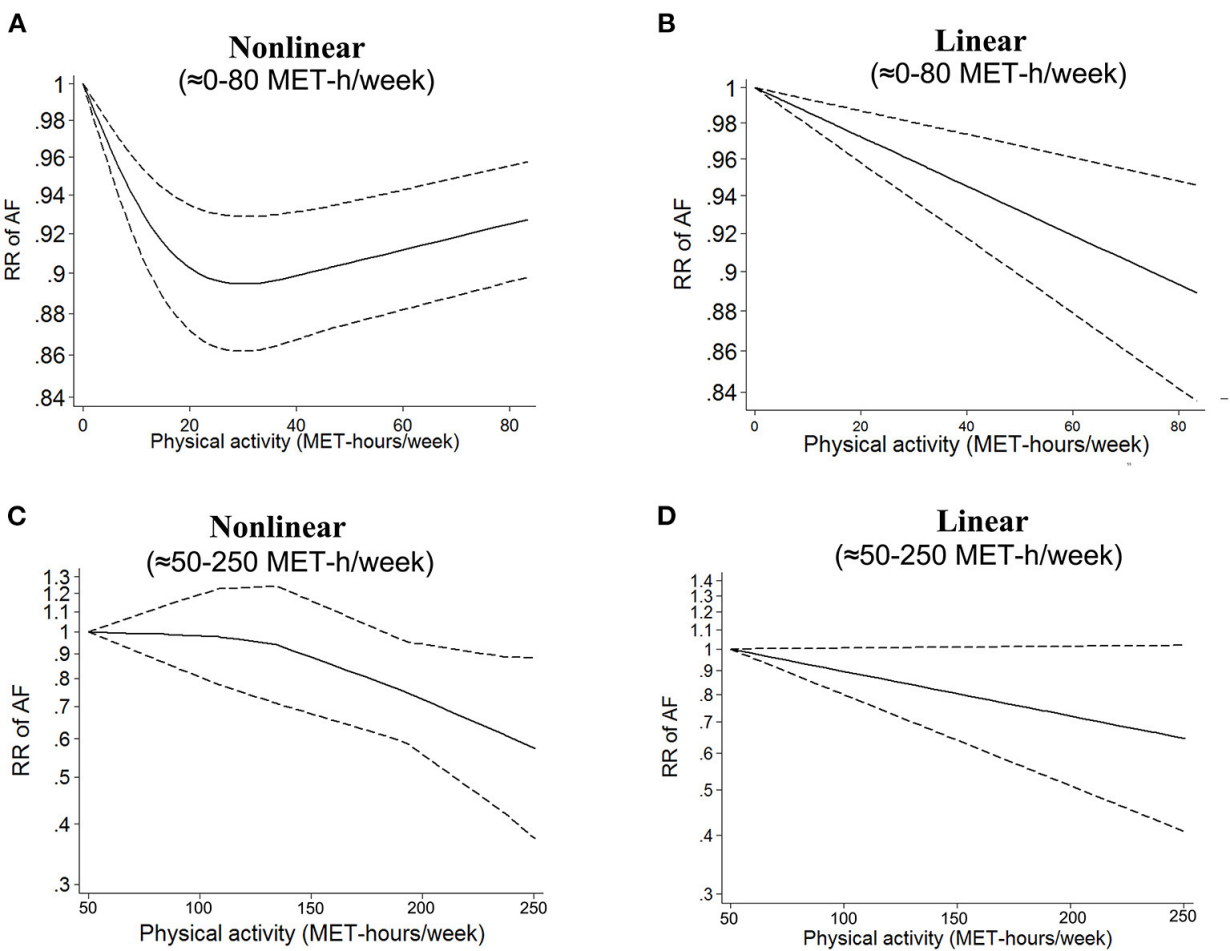
Dose–response analysis of physical activity and atrial fibrillation in the total population. **(A,B)** Non-linear and linear models of PA and AF in the range of 0–80 MET-h/week. **(C,D)** Non-linear and linear models of PA and AF at the high level of PA. The bold and dashed lines represent the estimated RR and 95% CI, respectively. The non-linear models were fit by using a restricted cubic spline. PA, physical activity; AF, atrial fibrillation; RR, risk ratio; CI, confidence interval.

The reference PA dose varied widely (from 0 to 68 MET-h/week) across the included studies, limiting the direct synthesis of all included studies in the dose–response analysis. Therefore, we first regarded low or sedentary activity (0–1.5 MET-h/week) as the reference (5–8, 20–30) to investigate AF risk of moderate PA dose compared with low or sedentary activity. As shown in Figure 2A and Supplementary Table 6, we found a significant non-linear relationship between PA in the range of 0–80 MET-h/week and AF risk (*I*^2^ = 0; *p*_non−linearity_ = 0.0016). The linear model applied to investigate the average relationship between PA and AF suggested that every 5 MET-h/week increment in PA was associated with a significantly decreased risk of AF (RR = 0.992, 95% CI: 0.988–0.996; *I*^2^ = 0; Figure 2B). In the sensitivity analyses, conducted by changing the assumptions of intensity or duration of PA exposure, the corresponding shapes of the dose–response curves were similar to that observed in the abovementioned primary analysis (Supplementary Figures 1, 2).

Subsequently, we included three studies (25, 30, 31) that regarded a moderate dose of PA (ranging from 38.5 to 63.5 MET-h/week) as the reference to investigate AF risk of a high PA dose (up to 250 MET-h/week) compared with a moderate PA dose. As shown in Figure 2C, there was a non-significant non-linear relationship between a high PA dose and AF risk (*I*^2^ = 0; *p*_non−linearity_ = 0.22). In the linear model, a 5 MET-h/week increase in PA was not associated with a change in AF risk (RR = 0.989, 95% CI: 0.964–1.014, *I*^2^ = 0, Figure 2D). Due to the small number of included studies in this part, the results should be interpreted with caution.

**Figure 3:**
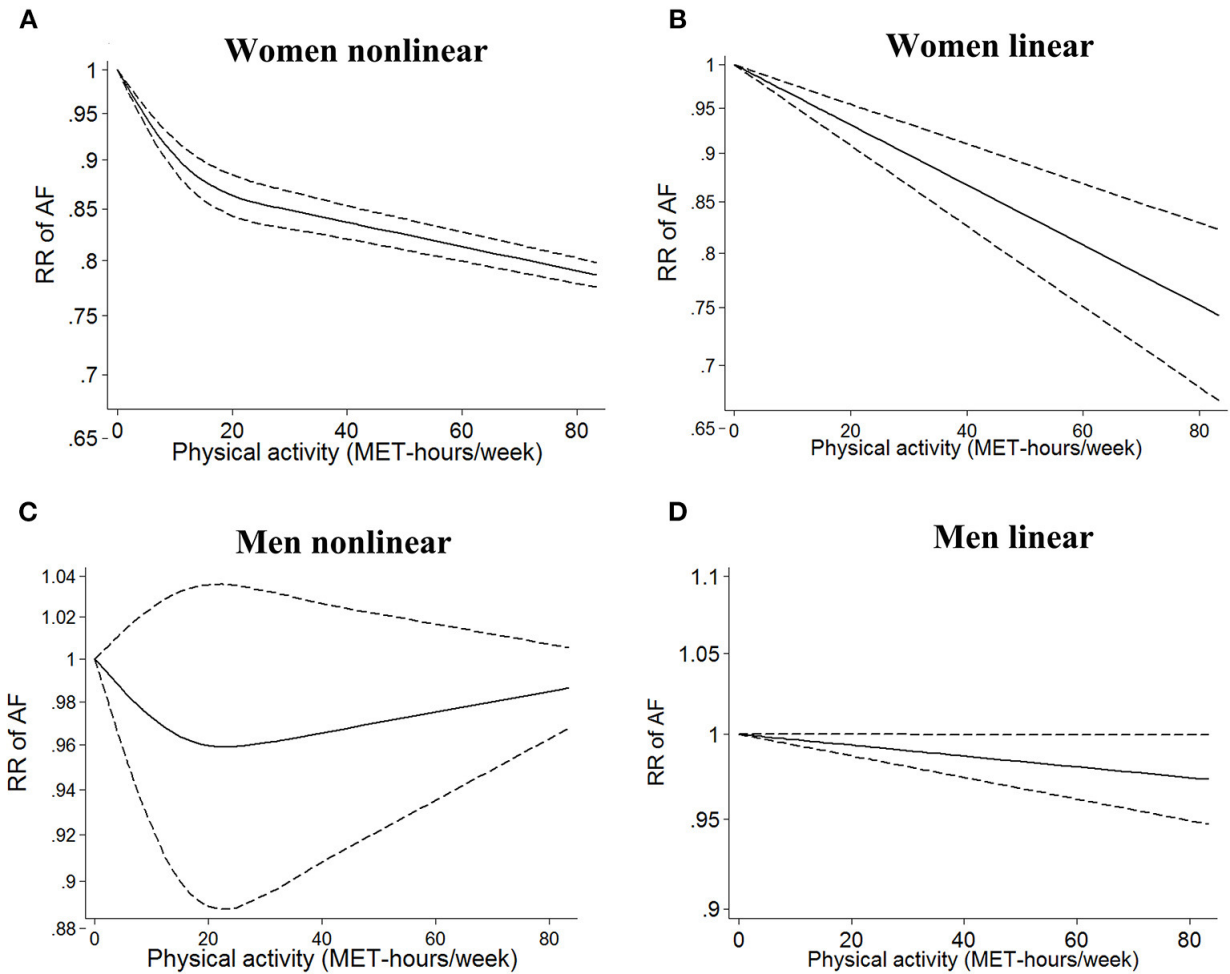
Dose–response analysis of physical activity and atrial fibrillation in the sex-stratified analysis. (**A,B)** Non-linear and linear models of PA and AF in females. **(C,D)** Non-linear and linear models of PA and AF in males. The bold and dashed lines represent the estimated RR and 95% CI, respectively. The non-linear models were fit by using a restricted cubic spline. PA, physical activity; AF, atrial fibrillation; RR, risk ratio; CI, confidence interval.

In the sex-stratified analysis, using low or sedentary activity as the reference, we found an inverse non-linear relationship between PA dose and AF risk in females (*I*^2^ = 90%, *p*_non−linearity_ < 0.0001, Figure 3A). The linear model showed that a 5 MET-h/week increase in PA (13) was associated with a 2.8% decrease in the risk of AF in females (RR = 0.982, 95% CI: 0.975–0.989, *I*^2^ = 71%, Figure 3B). In males, there was a non-significant non-linear relationship between PA dose and AF risk (*I*^2^ = 0%, *p*_non−linearity_ = 0.40, Figure 3C). The linear model showed that a 5 MET-h/week increase in PA was not associated with a change in AF risk (RR = 0.998, 95% CI: 0.994–1.002, *I*^2^ = 0, Figure 3D). There was a significant interaction between the two groups (*p*_interaction_ < 0.0001, Supplementary Figure 2).

We further examined the sex-stratified effect of PA level recommended by international institutions (32). In females, compared with sedentary activity, both the recommended basic PA level (150–300 min of moderate-intensity PA ≈ 11.25 MET-h/week; RR = 0.90; 95% CI: 0.88–0.92) and the strongly recommended PA level (>300 min of moderate-intensity PA ≈ >22.5 MET-h/week; RR = 0.86; 95% CI: 0.84–0.88) were associated with a decreased risk of AF. In males, decreased AF risk was not observed in the groups with recommended basic PA levels (RR = 0.96; 95% CI: 0.90–1.01) and strongly recommended PA levels (RR = 0.95; 95% CI: 0.88–1.02).

### Subgroup Analyses

In the age-stratified analysis, a 5 MET-h/week increase in PA was not associated with a change in AF risk (RR = 1.02, 95% CI: 0.95–1.09, *I*^2^ = 0) in the ≤ 50 years group, while a 5 MET-h/week increase in PA was associated with a decreased risk of AF (RR = 0.99, 95% CI: 0.98–0.99, *I*^2^ = 16%) in the >50 years subgroup (Table 2). Nevertheless, we observed no significant interaction between the two groups (*p*_interaction_ = 0.32). The exposure–effect analysis of the non-linear model yielded similar results to the linear model (Supplementary Figure 4).

**Table 2:** Subgroup analysis of physical activity and atrial fibrillation, exposure-effect analysis, per 5 MET h/week increment^#^ (8 MET-h/week is equivalent to 30 min of regular walking a day for 3 days a week).

**Items**		**Number of studies**	**RR (95% CI)**	***I*^**2**^ within each subgroup**	***P* for subgroup difference**
Result of primary analysis PA		15	0.992 (0.988–0.996)	0	–
Sex^※^	Females	9	0.982 (0.975–0.989)	71	< 0.0001
	Males	9	0.998 (0.994–1.002)	0	
Age^※^	≤ 50 years	5	1.020 (0.950–1.090)	0	0.32
	>50 years	10	0.992 (0.98–0.997)	16	
Measurement of PA	Questionnaire	14	0.992 (0.988–0.996)	0	–
	Objectively Measured	1	NA^*^	–	–
Region	Northern America	7	0.96 (0.93–0.99)	47	0.44
	Europe	6	0.994 (0.992–0.997)	73	
	Asia	2	NA^*^		
Follow-up duration	≤ 10 years	5	0.995 (0.991–0.998)	48	0.23
	>10 years	10	0.977 (0.957–0.997)	0	
AF Diagnosis	ECG or ICD	14	0.991 (0.986–0.997)	0	NA
	Others	1	NA^*^		
Sample size	< 100 000	11	0.97 (0.95–0.99)	0	0.31
	>100 000	4	0.994 (0.992–0.996)	94	

# *Subgroup of results that used the low or sedentary activity as a reference; the subgroup analysis that used moderate exercise as reference were not available due to the limited studies (N = 3)*.

* *Not available for dose-response analysis due to the limited data*.

※ *Multi-subgroup (age and sex) data were reported in some cohorts*.

*The number of studies is not always equal because the subgroup analyses were not available in several included studies*.

*AF, atrial fibrillation; PA, physical activity; NA, not available*.

In addition, we found no interactions in the other subgroup analyses based on the measurement of PA, region, follow-up duration, sample size, or AF diagnosis (all *p*_interaction_ > 0.05).

### Publication Bias

As shown in Supplementary Figure 5, there was no indication of publication bias according to Egger's test (*p* = 0.35), Begg's test (*p* = 0.42), or the funnel plot.

## Discussion

In the present study, our findings based on data from 16 prospective studies involving 1,449,017 individuals and 39,884 AF included the following: (1) an inverse non-linear association was found between PA level and incident AF in the general population, (2) a 5 MET-h/week increase in PA was associated with a reduced risk of AF in the linear model, and (3) the sex-stratified analysis indicated that the benefit of PA in reducing AF risk was predominantly in females.

Several studies regarding PA and the risk of AF have been published but have yielded inconsistent findings. Prior meta-analyses have reported that long-term endurance exercise is associated with an increased risk of AF among competitive athletes, whereas either total PA or intense PA has no impact on AF incidence in the general population. Neilson et al. (33) defined a “U”-shaped association of PA with AF risk. Subsequently, a J-shaped association was confirmed by a non-linear exposure–effect meta-regression analysis (34). Nevertheless, as proposed by Valenzuela et al. (3), the association between exercise and AF should be interpreted with caution because of the methodological limitations of existing evidence (4). On the one hand, some prior meta-analyses showed considerable heterogeneity in their study population because athletes were not excluded (35–37). As a result of cardiac adaptations to long-term endurance exercise, athletes often have a lower resting heart rate, larger diameter of the left atrium, elevated fibrosis level, and imbalance of autonomic function, potentially increasing susceptibility to AF (38). As such, sports-related AF among athletes should be disregarded when examining the relationship between PA and AF in the general population. On the other hand, given the different impacts of occupational PA and leisure-time PA on AF (38), it might not be reasonable to combine different types of PA for analysis in some meta-analyses (34, 39). Moreover, a newly published prospective cohort study by Elliott et al. found an inverse association between total PA (>500 MET-min/week) and AF risk in the general population (5). Considering the methodological limitations of previous studies and novel emerging evidence, the association of PA with AF risk warrants reevaluation. Unlike previous studies reporting a J-shaped association (34, 40), our current meta-analysis revealed an inverse relationship between PA level and AF risk in the general population. Notably, cardiorespiratory fitness is inversely associated with AF risk (41). Since PA, as one of the primary determinants of cardiorespiratory fitness, could improve fitness (42), the finding of similar benefits of PA and cardiorespiratory fitness in reducing AF risk is not unexpected.

A prior meta-analysis by Mohanty et al. revealed that moderate exercise was protective against AF regardless of sex (39). Our previous meta-analysis by Zhu et al. suggested that increasing PA was associated with a decreased risk of AF in females but an increased AF risk in males (3). In the newly published study by Elliott et al., PA was found to be associated with a reduced risk of incident AF across PA levels (from 500 to 5,000 MET-min/week) in females, whereas in males, a decreased risk of AF was observed for PA levels ranging from 500 to 1,500 MET-min/week, but a detrimental effect, enhancement of AF risk, was observed for PA levels of >5,000 MET-min/week (5). Elliott et al. further found that, in males, there was no association of vigorous PA at low to moderate doses with AF risk, but vigorous PA at extreme doses resulted in a 12% increased risk of AF; in contrast, a reduced incidence of AF across PA levels was observed in female participants (5). Therefore, whether such sex differences in the association between PA and AF risk exist in the general population remains unclear. To our knowledge, we are the first to assess the sex difference in the dose–response association between PA and AF risk in the general population, and we revealed a significant benefit of PA in decreasing AF risk in females. Consistent with this finding, a reduced risk of AF in females at recommended PA levels has been reported by international institutions (32). However, in males, we did not find a benefit of moderate or high levels of PA in AF. This result should be interpreted with caution. In the non-linear relationship shown in Figure 2C, a non-significant trend was found for the moderate level of PA, suggesting that moderate exercise might have some benefit. On the other hand, there was a trend of increased AF risk at high levels. This phenomenon does not surprise us, as it is consistent with some of our previous findings. It has long been suspected that the AF-promoting effects of exercise are predominantly expressed in males. The study of Elliott et al. (5) is the clearest and most definitive demonstration to date regarding potential sex differences. They showed that among women, only a protective effect of exercise was evident, expressed across the entire range of exercise levels. Among men, a clearly decreased AF risk was observed only with moderate PA; men became more AF prone with regular vigorous exercise.

The potential mechanism underlying the positive association between a high level of PA and AF in males in the general population is difficult to interpret. In endurance training athletes, compared with females, males exhibit more atrial electrophysiological changes (e.g., a larger atrial volume and left ventricle mass index, a greater relative wall thickness, and a longer p-wave duration) in response to rapid atrial pacing (43). Furthermore, in a frequency domain analysis of heart rate variability, male athletes were found to have a greater low-/high-frequency power ratio than females, suggesting greater sympathovagal balance in males. As several excellent reviews pointed out, multiple pathophysiological mechanisms might be responsible for the development of AF in athletes, such as atrial enlargement and fibrosis, atrial ectopic triggers, increased vagal tone, increased inflammation, and atrial function response to exercise (44–46). However, it remains unknown whether the discrepancy in atrial electrophysiological remodeling between the sexes could be responsible for the sex-specific exposure–effect relationship between PA and AF in the generally healthy population. The mechanistic basis of this phenomenon merits further investigation.

There is compelling evidence that individuals who have higher PA levels benefit from reduced incidences of all-cause mortality, diabetes, and cardiovascular diseases (32). Several international guidelines support the preventive effect of regular PA against non-communicable diseases such as cardiovascular diseases and diabetes (32). Among patients with established heart failure, the advancement of PA could reduce the risks of hospitalization and cardiovascular mortality and improve cardiorespiratory function and quality of life (47). In addition, a higher PA level is associated with a lower risk of AF in patients with heart failure (48). In patients with AF, a higher PA is associated with lower risks of all-cause mortality and cardiovascular mortality (49). Our current analysis extends the findings of previous studies, revealing an inverse association between PA and AF occurrence in the general population. In contrast, studies of long-term endurance exercise athletes have shown that high volumes of high-intensity PA are associated with an increased AF risk, thus indicating that “more is not always better” when referring to the association between PA and AF (50).

A previous meta-analysis showed that higher PA levels could increase AF risk in males (3). However, our current data showed that, in males, moderate-intensity PA of 150–300 min or >300 min did not significantly increase the risk of AF. We cannot exclude the possibility that if more patients are included in future studies, significant benefits of PA in reducing AF risk in males may be found. Collectively, our current evidence indicated that exercise at a dose in accordance with the recommended PA guidelines played a protective role in females, reducing AF risk among females, and was at least not harmful in males. The harmful dose of exercise was higher than the recommended PA levels. Therefore, both males and females could undergo an adequate amount of PA and maintain fitness with no fear of an increased AF risk.

### Strengths and Limitations

In this meta-analysis, we included high-quality studies with a large sample size of participants. We also excluded studies that enrolled athletes to focus on PA in the general population. A robust error meta-regression method was employed in the dose–response analysis to enhance the robustness of our results. In addition, we performed sensitivity analyses by changing assumptions regarding the intensity or duration of PA exposure; the results were similar to those of the primary analysis, suggesting the robustness of our findings. Nevertheless, our study has several limitations, as follows: First, although the included studies adjusted for several hybrid variables, residual confounding factors could not be excluded. Second, we calculated the PA dose based on assumptions regarding intensity or duration of PA exposure, which might have impacted our results. Third, nearly all the included studies assessed self-reported PA exposure, which might be prone to recall bias and overestimation. However, a recently published article showed an inverse association between PA and AF by using accelerometer measurements (51), which confirms our main results. Specifically, they found a weak correlation between accelerometer assessment and self-reported PA and a non-significant AF benefit from self-reported exercise. However, their results regarding self-reported PA were not consistent with previous studies based on the same dataset from the UK Biobank. Both Elliott et al. (5) and Said et al. (52) showed that greater self-reported activity may be beneficial with respect to AF risk. The discrepancy might derive from the small sample sizes and study differences in the methods of statistical analysis. As our results are based on a large sample, they may be reliable regardless of the form of assessment of PA. Fourth, studies investigating the effect of long-term endurance exercise on the risk of AF in athletes were excluded from our study. Further research could focus on the association of long-term endurance exercise with AF risk. Finally, although PA is the greatest determinant of fitness, PA and physical fitness have different definitions, and they may have independent associations with AF risk (42). Whether the relationship between PA and AF is independent of physical fitness needs further examination.

### Conclusions

The results from our dose–response meta-analysis revealed an inverse non-linear relationship between PA and AF risk in the general population. The beneficial effect of PA in reducing AF incidence was predominantly present in females.

## Data Availability Statement

The original contributions presented in the study are included in the article/Supplementary Material, further inquiries can be directed to the corresponding author/s.

## Author Contributions

WZ and XL performed the study design. QW and XL performed the selection, extraction, statistical analysis, and interpretation of the data. QW, YZ, and XL wrote and revised the manuscript. All authors contributed to the article and approved the submitted version.

## Funding

This study was funded by Young Teachers' Basic Scientific Research Business Expenses Project (WZ, 20ykpy72), China Postdoctoral Science Foundation (WZ, 2020M673016), and China National Postdoctoral Program for Innovative Talents (WZ, BX20200400).

## Conflict of Interest

The authors declare that the research was conducted in the absence of any commercial or financial relationships that could be construed as a potential conflict of interest.

## Publisher's Note

All claims expressed in this article are solely those of the authors and do not necessarily represent those of their affiliated organizations, or those of the publisher, the editors and the reviewers. Any product that may be evaluated in this article, or claim that may be made by its manufacturer, is not guaranteed or endorsed by the publisher.

## Abbreviations

PA: physical activity
AF: atrial fibrillation
MET: metabolic equivalent of task
RR: relative risk.
