# Supplementary material for: Sex-Specific Exposure–Effect Relationship Between Physical Activity and Incident Atrial Fibrillation in the General Population: A Dose–Response Meta-Analysis of 16 Prospective Studies
Source: Front Cardiovasc Med. 2021 Sep 22;8:710071. doi: 10.3389/fcvm.2021.710071 (PMC8492974; doi:10.3389/fcvm.2021.710071)
Supplement: Supplementary file 1 [file Data_Sheet_1.docx]

**SUPPLEMENTAL MATERIAL**

**Supplemental Table S1. Summary of PA exposure dose for the included studies**

| **Author, year, country** | **PA exposure (dose^*^ in MET h/wk)** | **RRs (95%CI)** |
| --- | --- | --- |
| Lee et al,2020, Korea | Sedentary PA (0)  Insufficient PA (5)  Sufficient PA (17.5)  Health-enhancing PA (37.5) | Reference  1.00(0.74–1.37)  1.34(0.91–1.97)  1.27(0.72–2.23) |
| Choi et al, 2019, Korea | <11.3 MET h/day (48.2)  11.3–19.4 MET h /day (106.61)  19.5–35.5 MET h /day (192.5)  ≥35.6 MET h /day (235.2) | Reference  0.78 (0.51–1.19)  0.58 (0.37–0.90)  0.88 (0.57–1.36) |
| Jin et al, 2019, Korea | None (0)  1-500 MET min/wk (4.1)  500-1000 MET min/wk(12.5)  ≥1000 MET min/wk (25) | Reference  0.94 (0.86–1.03)  0.88 (0.80–0.97  0.93 (0.85–1.03) |
| Elliott et al, 2019, UK | Men  0 MET-min/wk (0)  500 MET-min/wk (8.3)  1000 MET-min/wk(16.6)  1500 MET-min/wk (25)  2000 MET-min/wk (33.3)  2500 MET-min/wk (41.6)  5000 MET-min/wk (83.3)  Women  0 MET-min/wk (0)  500 MET-min/wk (8.3)  1000 MET-min/wk(16.6)  1500 MET-min/wk (25)  2000 MET-min/wk (33.3)  2500 MET-min/wk (41.6)  5000 MET-min/wk (83.3) | Reference  0.95 (0.91-1.0)  0.92(0.85-1.0)  0.90(0.82-1.0)  0.91(0.82-1.0)  0.91(0.82-1.0)  0.98(0.90-1.06)  0.94(0.88-1.0)  Reference  0.88(0.79-0.99)  0.85(0.74-0.98)  0.83(0.72-0.96)  0.82(0.72-0.94)  0.80(0.71-0.91) |
| Albrecht et al,2018,USA | Total PA  38.7 MET h/wk  74.7 MET h/wk  123.2 MET h/wk  Sports  0 MET h/wk  5.5 MET h/wk  19.8 MET h/wk | Reference  1.08(0.91, 1.29)  1.05(0.87, 1.25)  Reference  1.02(0.85-1.23)  1.05(0.87-1.27) |
| O’Neal et al,2020, USA | 0.7 min/day (0)  4.4 min/day (2.3)  13 min/day (5.3)  40 min/day (16.3) | Ref  0.77 (0.58, 1.01)  0.72 (0.53, 0.98)  0.62 (0.44, 0.87) |
| Mokhayeri et al, 2017, USA | Man  Total PA  5-122 MET h/wk(63.5)  123-173 MET h/wk(148)  174-233 MET h/wk(203.5)  <234 MET h/wk(264)  Intentional PA  None (0)  1-14 MET h/wk (7.5)  15-29 MET h/wk (23)  ≥30 MET h/wk (37.5)  Woman  Total PA  5-122 MET h/wk(63.5)  123-173 MET h/wk(148)  174-233 MET h/wk(203.5)  <234 MET h/wk(264)  Intentional PA  None (0)  1-14 MET h/wk (7.5)  15-29 MET h/wk (23)  ≥30 MET h/wk (37.5) | Reference  0.77(0.38-1.54)  0.57(0.26-1.25)  0.59(0.29-1.28)  Reference  1.28 (0.59, 2.77)  1.02 (0.42, 2.44)  1.27(0.57, 2.83)  Reference  0.45(0.18-1.08)  0.33(0.10-1.11)  0.37(0.14-1.00)  Reference  0.69 (0.29, 1.65)  0.54 (0.18, 1.63)  0.53 (0.17, 1.66) |
| Morseth et al, 2016, Norway | Low activity (3) Moderate activity (20) High activity (22.5)  Vigorous activity (24) | Reference 0.81 (0.68, 0.96)  0.94 (0.75, 1.19)  1.29 (0.73, 2.30) |
| Skielboe et al, 2016, Denmark | Low activity (0) Moderate activity (9) High activity (20)  Very high activity (40) | 0.92 (0.77, 1.09) Reference 1.05 (0.92, 1.20)  1.16 (0.80, 1.67) |
| Drca et al, 2015, Sweden | <1 h/wk (4)  1 h/wk (8)  2-3 h/wk (20)  ≥4 h/wk (36) | Reference  0.89 (0.79, 1.00)  0.92 (0.82, 1.02)  0.85 (0.75, 0.95) |
| Azarbal et al,  2014, USA | No activity (0)  0-3 MET h/wk (1.5)  3-9 MET h/wk (6)  >9 MET h/wk (12) | Reference 0.98 (0.91, 1.06) 0.94 (0.88, 1.01)  0.90 (0.85, 0.96) |
| Drca et al,  2014, Sweden | age 30  never (0)  <20 min/day (4.6)  20–40 min/day (10.5)  40-60 min/day (17.4)  >60 min/day (32.6) | Reference  1.08 (0.0.92,1.26)  0.98 (0.84, 1.15)  1.03 (0.88, 1.21)  1.08 (0.93, 1.27) |
| Huxley et al, 2014, USA | Poor (0)  Intermediate (5)  Ideal (5.6) | Reference  0.95 (0.84, 1.07)  0.89 (0.80, 1.00) |
| Thelle et al, 2013, Norway | Women  Sedentary (0)  Moderate (20)  Intermediate (22.5)  Men  Sedentary (0)  Moderate (20)  Intermediate (22.5)  Intensive (24) | Reference 0.96 (0.71, 1.29) 0.87 (0.55, 1.38)  Reference  1.12 (0.88, 1.42)  1.36 (1.05, 1.77)  3.14 (2.17, 4.54) |
| Everett et al, 2011, USA | <2.0 MET h/wk (0.6)  2-5.9 MET h/wk (4)  5.9-12 MET h/wk (8)  12-23 MET h/wk (16.5)  ≥23 MET h/wk (28.75) | Reference  1.12 (0.92, 1.37)  1.01 (0.82, 1.24)  1.01 (0.82, 1.24)  1.00 (0.81, 1.25) |
| Mozaffarianet et al, 2008, USA | <35 kcal/wk (0.57)  35-404 kcal/wk (4.9)  405-885kcal/wk (13.2)  890-1838kcal/wk (27.9)  >1840kcal/wk (47) | Reference  0.86 (0.71, 1.03)  0.75 (0.61, 0.90)  0.78 (0.65, 0.95)  0.64 (0.52, 0.79) |

^*^Full details of MET/h dose assignment are in **Table S3**

**Supplemental Table S2. List of included studies in this meta-analysis**

| [1] Mozaffarian D, Furberg CD, Psaty BM, Siscovick D. Physical Activity and Incidence of Atrial Fibrillation in Older Adults: The Cardiovascular Health Study. Circulation. 2008;118:800-7. |
| --- |
| [2] Thelle DS, Selmer R, Gjesdal K, Sakshaug S, Jugessur A, Graff-Iversen S, et al. Resting heart rate and physical activity as risk factors for lone atrial fibrillation: a prospective study of 309,540 men and women. Heart. 2013;99:1755-60. |
| [3] Huxley RR, Misialek JR, Agarwal SK, Loehr LR, Soliman EZ, Chen LY, et al. Physical Activity, Obesity, Weight Change, and Risk of Atrial Fibrillation: The Atherosclerosis Risk in Communities Study. Circulation: Arrhythmia and Electrophysiology. 2014;7:620-5. |
| [4] Mokhayeri Y, Hashemi-Nazari SS, Mansournia MA, Soori H, Khodakarim S. The association between physical activity and atrial fibrillation applying the Heaviside function in survival analysis: the Multi-Ethnic Study of Atherosclerosis. Epidemiology and Health. 2017;39:e2017024. |
| [5] Albrecht M, Koolhaas CM, Schoufour JD, van Rooij FJ, Kavousi M, Ikram MA, et al. Physical activity types and atrial fibrillation risk in the middle-aged and elderly: The Rotterdam Study. Eur J Prev Cardiol. 2018;25:1316-23. |
| [6] Choi YW, Park M, Lim YH, Myung J, Kim BS, Lee Y, et al. Independent effect of physical activity and resting heart rate on the incidence of atrial fibrillation in the general population. Sci Rep. 2019;9:11228. |
| [7] Jin MN, Yang PS, Song C, Yu HT, Kim TH, Uhm JS, et al. Physical Activity and Risk of Atrial Fibrillation: A Nationwide Cohort Study in General Population. Sci Rep. 2019;9:13270. |
| [8] Lee SH, Ryu S, Lee JY, Seo DC, Kim BJ, Sung KC. Association between self-reported physical activity and incident atrial fibrillation in a young Korean population. Sci Rep. 2019;9:4222. |
| [9] Elliott AD, Linz D, Mishima R, Kadhim K, Gallagher C, Middeldorp ME, et al. Association between physical activity and risk of incident arrhythmias in 402 406 individuals: evidence from the UK Biobank cohort. Eur Heart J. 2020;41:1479-86. |
| [10] Everett BM, Conen D, Buring JE, Moorthy MV, Lee IM, Albert CM. Physical activity and the risk of incident atrial fibrillation in women. Circ Cardiovasc Qual Outcomes. 2011;4:321-7. |
| [11] O'Neal WT, Bennett A, Singleton MJ, Judd SE, Howard G, Howard VJ, et al. Objectively Measured Physical Activity and the Risk of Atrial Fibrillation (from the REGARDS Study). Am J Cardiol. 2020;128:107-12. |
| [12] Drca N, Wolk A, Jensen-Urstad M, Larsson SC. Atrial fibrillation is associated with different levels of physical activity levels at different ages in men. Heart. 2014;100:1037-42. |
| [13] Azarbal F, Stefanick ML, Salmoirago-Blotcher E, Manson JE, Albert CM, LaMonte MJ, et al. Obesity, physical activity, and their interaction in incident atrial fibrillation in postmenopausal women. J Am Heart Assoc. 2014;3. |
| [14] Morseth B, Graff-Iversen S, Jacobsen BK, Jorgensen L, Nyrnes A, Thelle DS, et al. Physical activity, resting heart rate, and atrial fibrillation: the Tromso Study. Eur Heart J. 2016;37:2307-13. |
| [15] Skielboe AK, Marott JL, Dixen U, Friberg JB, Jensen GB. Occupational physical activity, but not leisure-time physical activity increases the risk of atrial fibrillation: The Copenhagen City Heart Study. European Journal of Preventive Cardiology. 2016;23:1883-93. |
| [16] Drca N, Wolk A, Jensen-Urstad M, Larsson SC. Physical activity is associated with a reduced risk of atrial fibrillation in middle-aged and elderly women. Heart. 2015;101:1627-30. |

**Supplemental Table S3. Summary of metabolic equivalent of task (MET) h/week assignment calculations for included studies**

| **Study, year,** | **PA type** | **Frequency** | **Duration** | **Intensity** | **Assigned MET h/wk** | **Additional information** |
| --- | --- | --- | --- | --- | --- | --- |
| Mokhayeri et al, 2017 | Total PA and LTPA |  |  | **Woman**  Total PA  5-122 MET h/wk  123-173 MET h/wk  174-233 MET h/wk  <234 MET h/wk  Intentional PA  0 MET h/wk  1-14 MET h/wk  15-29 MET h/wk  ≥30 MET h/wk  **Man**  Total PA  5-122 MET h/wk  123-173 MET h/wk  174-233 MET h/wk  <234 MET h/wk  Intentional PA  0 MET h/wk  1-14 MET h/wk  15-29 MET h/wk  ≥30 MET h/wk | 63.5  148  203.5  264  0  7.5  23  37.5  63.5  148  203.5  264  0  7.5  23  37.5 |  |
| Morseth et al, 2016 | LTPA |  | Low activity (reading, watching TV, or other sedentary activities) |  | 0 |  |
|  |  |  | Moderate activity (walking, cycling, or other forms of exercise at least 4h a week) (5 h/wk) | MPA=4 MET  (3 marginal METs in sensitive analysis) | 20  (12) |  |
|  |  |  | High activity (participation in recreational sports, heavy gardening, etc. at least 4h a week) (5 h/wk) | MVPA=4.5 MET  (3.5 marginal METs in sensitive analysis) | 22.5  (17.5) |  |
|  |  | several times a week (4 times) | Vigorous activity (participation in hard training or sports competitions regularly several times a week)  (45 min/session) (30 min/session) | VPA=8 MET  (7 marginal METs in sensitive analysis) | 42  (34.6)(28) |  |
| Skielboe et al, 2016 | LTPA |  | Entirely sedentary (reading, watching television or movies) |  | 0 |  |
|  |  |  | Light physical activity for 2-4 h/week (3 h/wk) | LPA=3 MET  (2 marginal METs in sensitive analysis) | 9  (6) |  |
|  |  |  | Light physical activity for more than 4 h/week (e.g. brisk walking, fast biking, heavy gardening) (5 h/wk) | MPA=4 MET  (3 marginal METs in sensitive analysis) | 20  (15) |  |
|  |  |  | Highly vigorous physical activity for more than 4 h/wk (5 h/wk) | VPA=8 MET  (7 marginal METs in sensitive analysis) | 40  (35) |  |
| Drca et al, 2015 | LTPA |  | Walking/bicycling (min/dya)  Almost never |  | 0 |  |
|  |  |  | <20 | 10 | 7.5 |  |
|  |  |  | 20–40 | 30 | 14 |  |
|  |  |  | >40 | 50 | 28 |  |
| Azarbal et al, 2014 | Total PA |  |  | No activity  0-3 MET h/wk  3-9 MET h/wk  >9 MET h/wk | 0  1.5  6  12 |  |
| Drca et al, 2014 | LTPA |  | walking/bicycling-min/day (mean age 60) (baseline)  Never (0 h/wk)  <20 min/day (1.1 h/wk)  20-40 min/day (3.5 h/wk)  40-60 min/day ((5.8 h/wk))  >60 min/day (8.1 h/wk) | MPA=4 MET  (3 marginal METs in sensitive analysis) | 0  4.6(3.3)  14(10.5)  23.3(17.4)  32.6(24.3) |  |
| Huxley et al, 2014 | LTPA |  | Poor (no moderate or vigorous) |  | 0 |  |
|  |  |  | Intermediate (1–149 min/wk moderate) (1.25 h/wk) | MPA=4 MET  (3 marginal METs in sensitive analysis) | 5(3.75) |  |
|  |  |  | Ideal (1–149 min/wk moderate + vigorous) (1.25 h/wk) | MVPA=4.5 MET  (3.5 marginal METs in sensitive analysis) | 5.6(4.375) |  |
| Thelle et al, 2013 | LTPA |  | Sedentary (reading, watching TV, or other sedentary activity) |  | 0 |  |
|  |  |  | Moderate (walking, cycling, or other forms of exercise at least 4 h per week (5 h/wk) | MPA=4 MET  (3 marginal METs in sensitive analysis) | 20(15) |  |
|  |  |  | Intermediate (participation in recreational sports, heavy gardening or similar activities for at least 4 h per week) (5 h/wk) | MVPA = 4.5 MET (3.5 marginal METs in sensitive analysis) | 22.5(17.5) |  |
|  |  |  | Intensive (Participation in hard training or sports competitions, regularly and several times per week)(45 minutes/times) (30 min/session) | VPA=8 MET  (7 marginal METs in sensitive analysis) | 42(34.6)(28) |  |
| Everett et al, 2011 | LTPA |  |  | <2.0 MET h/wk  2-5.9 MET h/wk  5.9-12 MET h/wk  12-23 MET h/wk  ≥24 MET h/wk | 1.6  4  8  16.5  28.75 |  |
| Mozaffarianet et.al, 2008 | LTPA |  |  | <35 kcal/wk  (28 kcal/wk)  35–404 kcal/wk (219.5 kcal/wk)  405–885 kcal/wk (645 kcal/wk)  890–1838 kcal/wk (1364 kcal/wk)  >1840 kcal/wk (2300 kcal/wk) | 0.57  4.9  13.2  27.9  47 | transformation between kcal/week (Y) and MET-h/week (X) using the following formula:  (4.5 [MET] * 2.5 [h])/550 [kcal] (for =X [MET∗h]/Y [kcal] |
| Lee et al, 2019 |  |  |  | Sedentary PA (0)  Insufcient PA (0-600)  At least 600 MET-minutes/week"(600-1500)  1500-3000MET-min/week (1500-3000) | 0  5  17.5  37.5  37.5 |  |
| Choi et al ,2018 |  |  |  | 0-11.3 METs-h/day (5.6)  11.3–19.4 METs-h/day (15.3)  19.5–35.5 METs-h/day (27.5)  ≥35.6 METs-h/day (43.6) | 48.23  106.6  192.5  192.5  235.2 |  |
| Elliott et al,2020 |  |  |  | 0 MET-mins/wk  500 MET-mins/wk  1000 MET-mins/wk  1500 MET-mins/wk  2000 MET-mins/wk  2500 MET-mins/wk  5000 MET-mins/wk | 0  8.333333333  16.66666667  25  33.33333333  41.66666667  83.33333333 |  |
| Albrecht et al,2018 |  |  |  |  | Total PA  38.7  74.7  123.2  Sport related PA  0  5.5  19.8 |  |
| O’Neal,2020 |  |  | Q1-0.7 min/day  Q2-4.4 min/day  Q3-13 min/day  Q4-40 min/day | MVPA=4.5 MET-h/week  (3.5 marginal METs in sensitive analysis) | 0(0)  2.3(1.79)  6.8(5.3)  21(16.3) |  |

MET h/wk doses were assigned from descriptions identified within the individual studies.
wk: week; EE: energy expenditure (in kcal); MET: metabolic equivalent of task; MET unit accounting for the individual basal metabolic rate; PA: physical activity; LTPA: light physical activity; MPA: moderate physical activity; MVPA: moderate to vigorous physical activity; VPA: vigorous physical activity; Black font: Reported PA duration, frequency or intensity; Red: Assumed; Green: Calculated.

**Supplemental Table S4. Quality assessment of the included studies**

| **Author**  **(Publication Year)** | **Newcastle-Ottawa Scale** | | | | | | | | | |
| --- | --- | --- | --- | --- | --- | --- | --- | --- | --- | --- |
|  | **Selection** | | | **Comparability** | | | **Outcome** | | | **Total** |
|  | **a** | **b** | **c** | **d** | **e** | **f** | **g** | **h** | **i** |  |
| Albrecht-2019 | 1 | 1 | 1 | 1 | 1 | 1 | 1 | 1 | 1 | 9 |
| Lee et al,2020 | 1 | 1 | 1 | 1 | 1 | 1 | 1 | 1 | 0 | 8 |
| Choi et al, 2019 | 1 | 1 | 1 | 1 | 1 | 1 | 1 | 1 | 0 | 8 |
| Jin et al, 2019 | 1 | 1 | 1 | 1 | 1 | 1 | 1 | 1 | 1 | 9 |
| Elliott et al, 2019 | 1 | 1 | 1 | 1 | 1 | 1 | 1 | 1 | 1 | 9 |
| O’Neal et al, 2018 | 1 | 1 | 1 | 1 | 1 | 1 | 1 | 1 | 0 | 8 |
| Mokhayeri-2017 | 1 | 1 | 1 | 1 | 0 | 1 | 1 | 1 | 0 | 7 |
| Morseth-2016 | 1 | 1 | 1 | 1 | 1 | 1 | 1 | 1 | 1 | 9 |
| Skielboe-2016 | 1 | 1 | 1 | 1 | 1 | 1 | 1 | 1 | 1 | 9 |
| Drca-2015 | 0 | 1 | 1 | 1 | 1 | 1 | 1 | 1 | 1 | 8 |
| Azarbal-2014 | 0 | 1 | 1 | 1 | 1 | 1 | 1 | 1 | 0 | 7 |
| Drca-2014 | 0 | 1 | 1 | 1 | 1 | 1 | 1 | 1 | 0 | 7 |
| Huxley-2014 | 1 | 1 | 1 | 1 | 1 | 1 | 1 | 1 | 1 | 9 |
| Thelle-2013 | 1 | 1 | 1 | 1 | 1 | 1 | 1 | 0 | 0 | 7 |
| Everett-2011 | 0 | 1 | 1 | 1 | 1 | 1 | 1 | 1 | 1 | 8 |
| Mozaffarian-2008 | 1 | 1 | 1 | 1 | 1 | 1 | 1 | 1 | 0 | 8 |

1. Representativeness of the exposed cohort.
2. Selection of the non-exposed cohort.
3. Ascertainment of exposure.
4. Demonstration that outcome of interest was not present at start of study.
5. Comparability of cohorts on the basis of the design or analysis (adjusted for age).
6. Comparability of cohorts on the basis of the design or analysis (adjusted for any other factor).
7. Assessment of outcome.
8. Was follow-up long enough for outcomes to occur. (>5 years).
9. Adequacy of follow-up of cohorts.

**Supplemental Table S5**. **Relative risks between PA and AF, from the nonlinear dose-response analysis (0-80 MET-h/week)**

| **Outcome**  **PA(MET-h/week)** | AF incidence |
| --- | --- |
| **PA** | RR (95% CI) |
| 0 | Ref |
| 1.5 | 0.99 (0.99-1.00) |
| 2.7 | 0.98 (0.97-0.99) |
| 4.6 | 0.97 (0.96-0.99) |
| 6 | 0.96 (0.94-0.98) |
| 8 | 0.95 (0.93-0.98) |
| 10 | 0.94 (0.91-0.97) |
| 14 | 0.93 (0.89-0.96) |
| 17.5 | 0.92 (0.88-0. 94) |
| 20 | 0.90 (0.87-0. 93) |
| 25 | 0.90 (0.86-0. 93) |
| 30 | 0.90 (0. 87-0. 93) |
| 40 | 0.90 (0.87-0.93) |
| 83 | 0.93 (0.90-0.97) |
| P for nonlinear | <0.0001 |

AF: atrial fibrillation; PA: physical activity


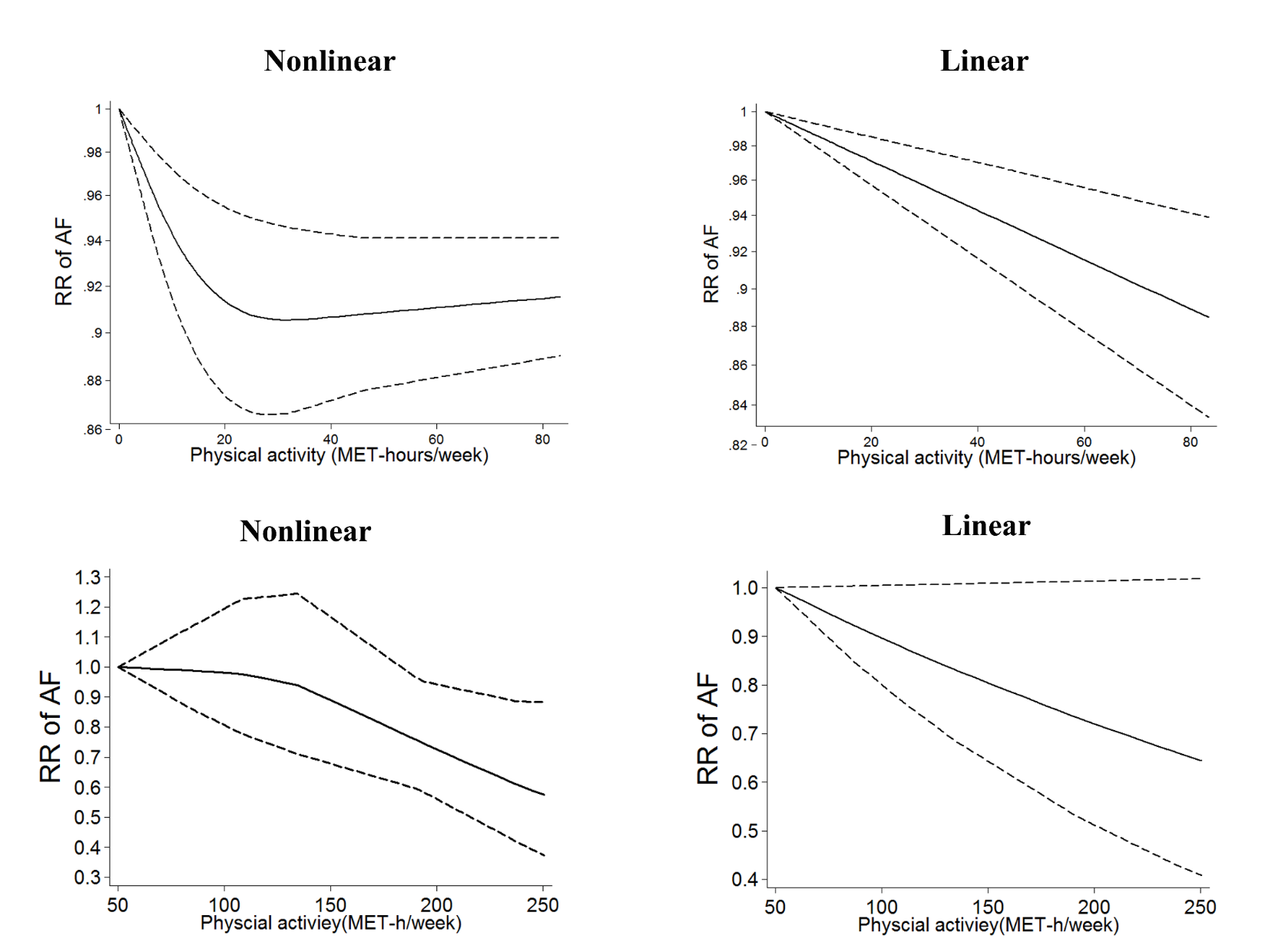


**Supplemental Figure S1. Sensitivity analysis by changing intensity assumption between physical activity and AF (linear model or non-linear model)**

**(A and B. By pooling studies of studies** which used low or sedentary physical activity (0-80 MET-h/week) as reference**; C and D.** By pooling studies of studies which used moderate physical activity (38-264 MET-h/week). **(intensity assumption applied as LPA = 2 MMET, MPA = 3 MMET, MVPA = 3.5 MMET, and VPA = 7 MMET).** Nonlinear model was fit by using restricted cubic spline. The bold and the dashed lines represent the estimated RR and 95% CI, respectively. AF: atrial fibrillation; MVPA: moderate to vigorous PA; LPA: light PA; VPA: vigorous PA. MPA: moderate PA

**
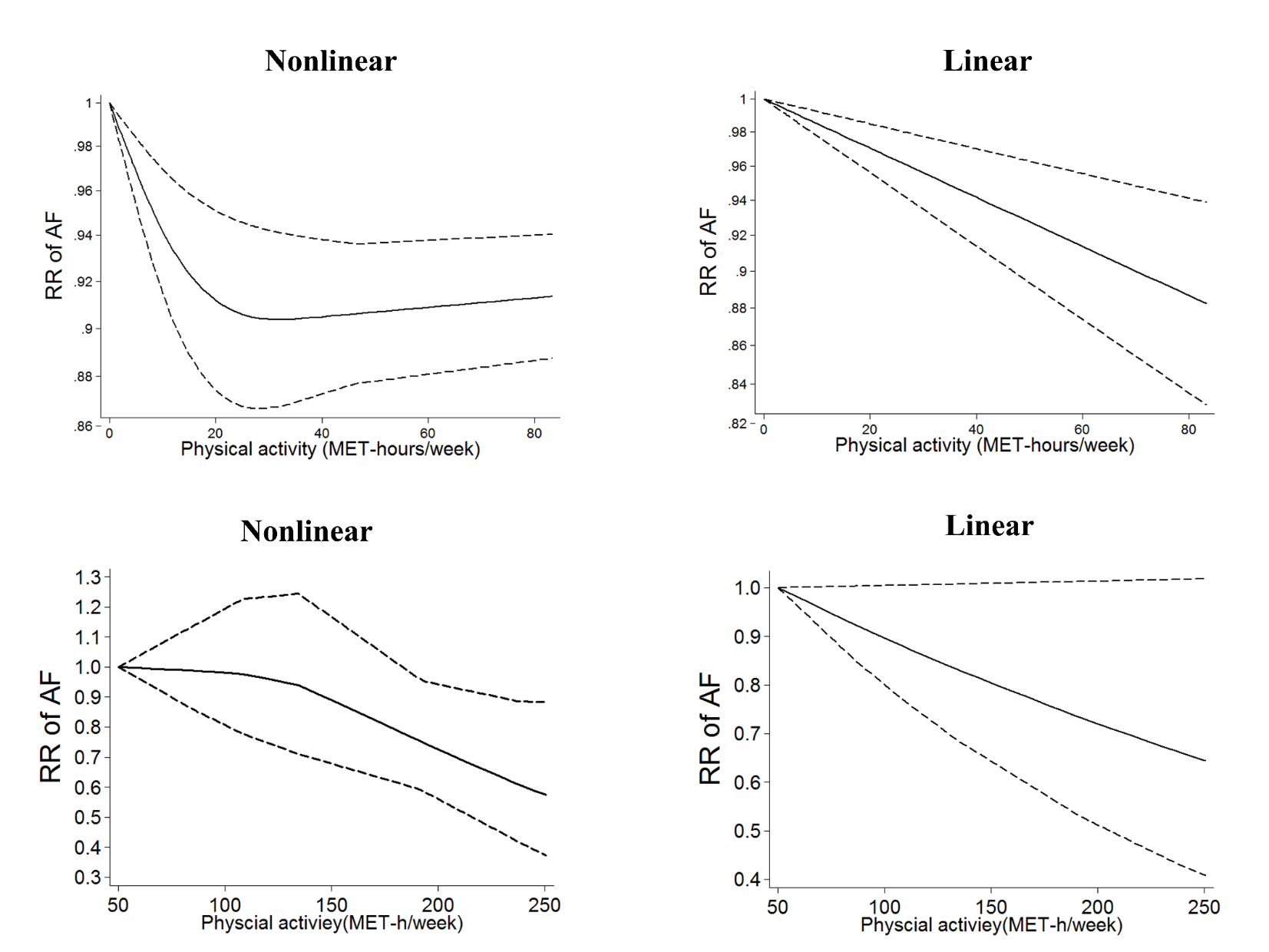
**

**Supplemental Figure S2. Sensitivity analysis by changing assumption of 45 min to 30 min between physical activity (PA) and AF (linear model).**

**(A and B. By pooling studies of studies** which used low or sedentary physical activity (0-80 MET-h/week) as reference**; C and D.** By pooling studies of studies which used moderate physical activity (38-263 MET-h/week) as reference). AF: atrial fibrillation; MVPA: moderate to vigorous PA; LPA: light PA; VPA: vigorous PA. MPA: moderate PA; Nonlinear model was fit by using restricted cubic spline. The bold and the dashed lines represent the estimated RR and 95% CI, respectively.


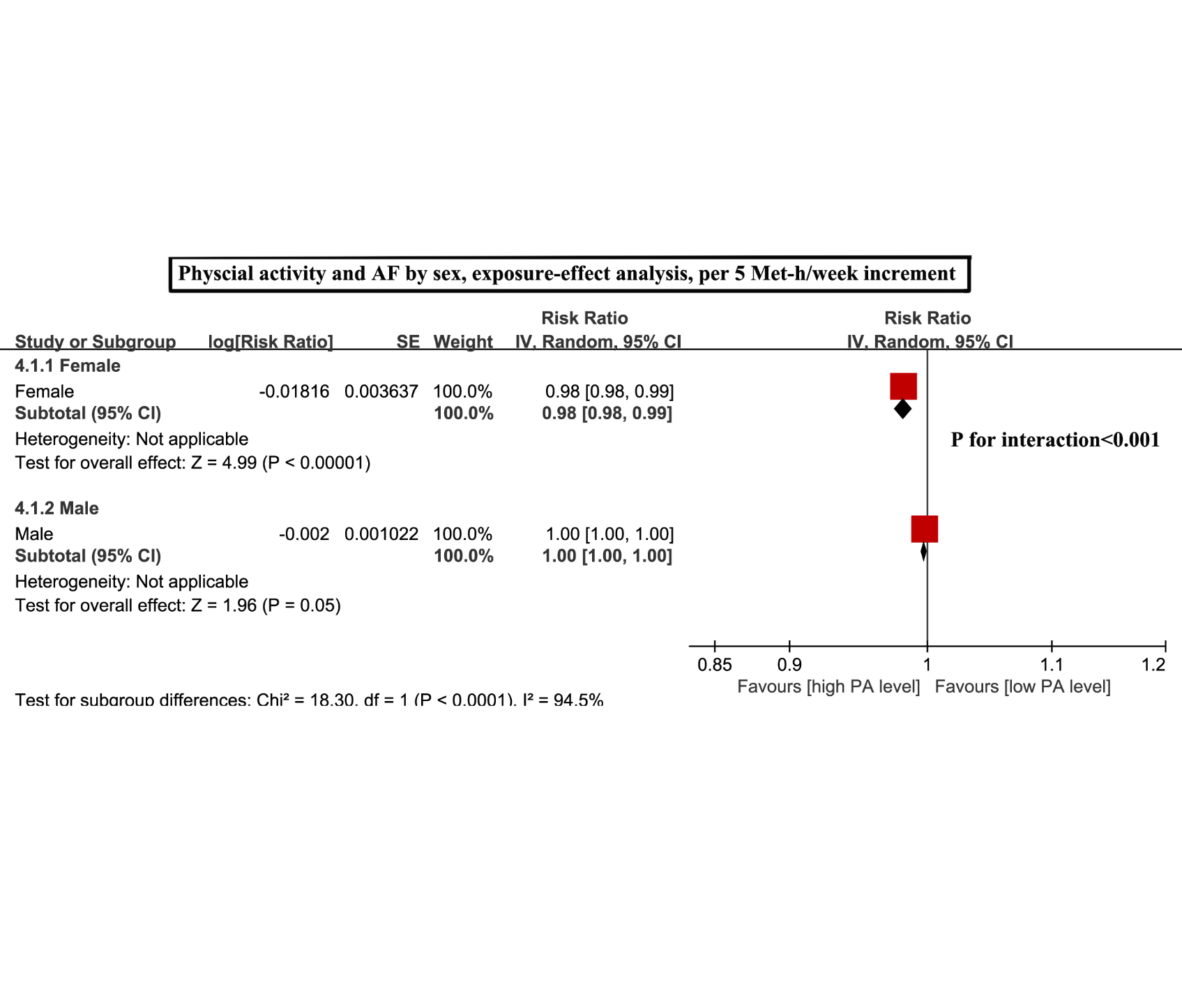


**Supplemental Figure S3. physical activity and AF, by sex, exposure-effect analysis, per 5 MET-h/week increment.**

AF: atrial fibrillation; PA: physical activity


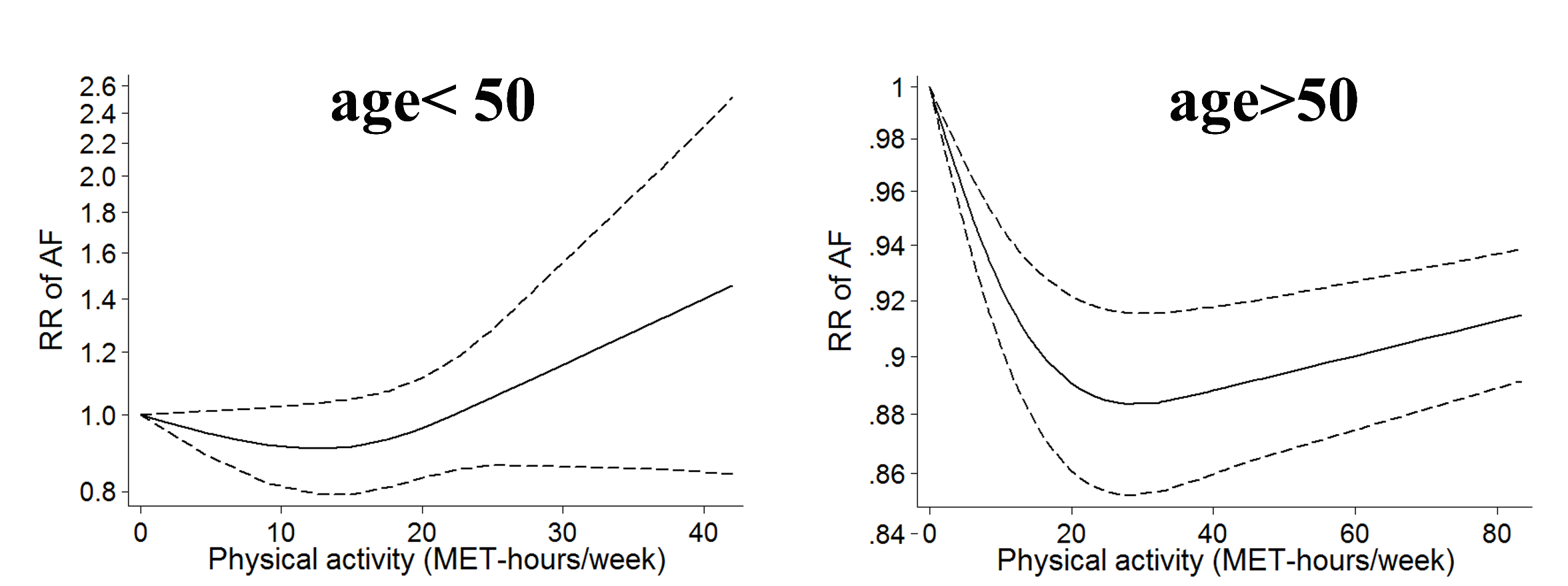


**Supplemental Figure S4. Physical activity and AF (non-linear) by age***

*By pooling studies of studies which reported physical activity range 0 from 80 MET-h/week, the pooling analysis that used moderate exercise as reference were not available due to the limited studies(N=3). Nonlinear model was fit by using restricted cubic spline.

**
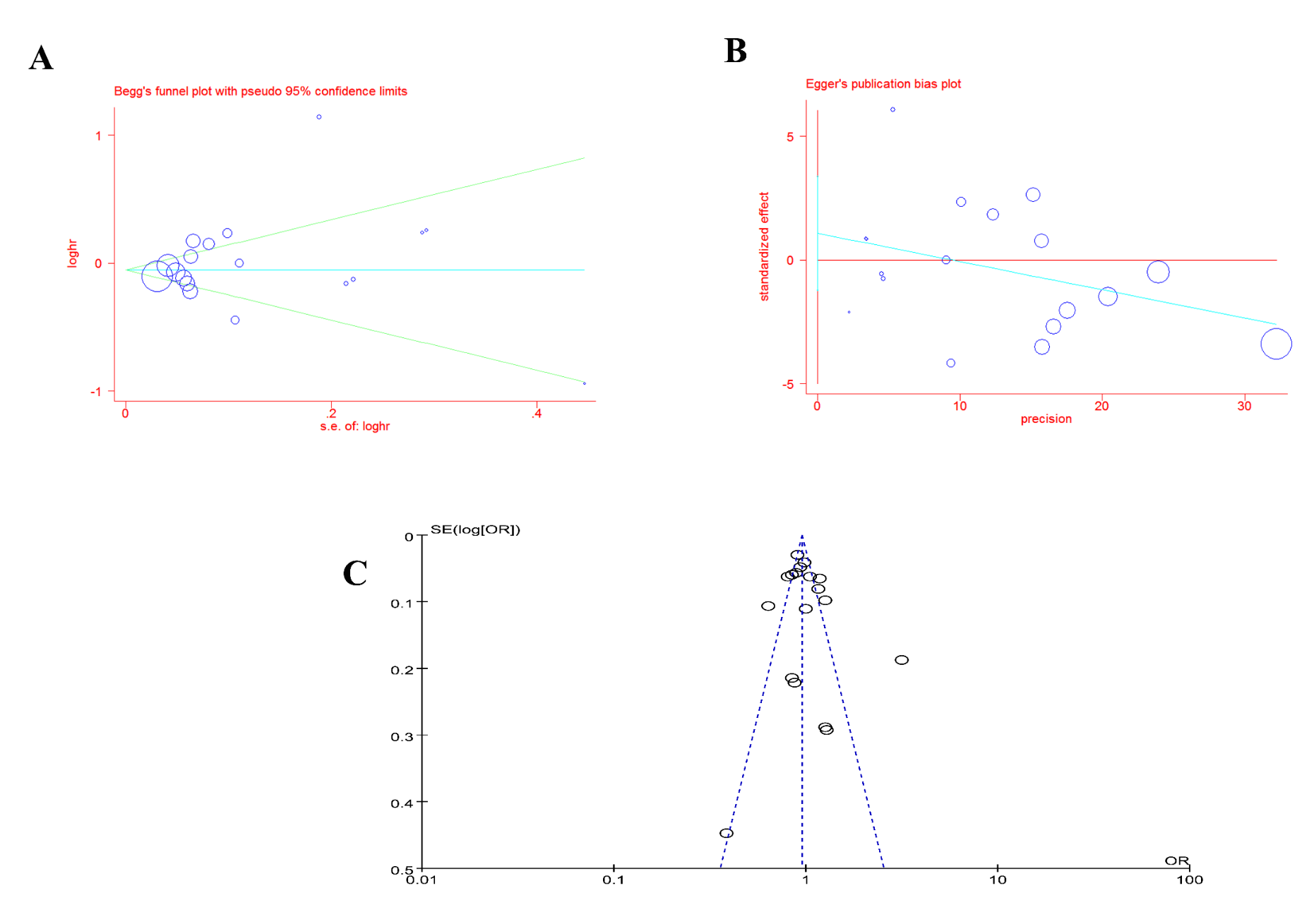
**

**Supplemental Figure S5. Publication analysis (highest versus lowest PA categories) of all included studies for PA. Begger’s test (A), Egger’s test (B), funnel plot(C).** PA: physical activity
